# Risk factors for developmental quotients in ASD children: A cross-sectional study

**DOI:** 10.3389/fpsyg.2023.1126622

**Published:** 2023-03-13

**Authors:** Han-Yu Dong, Chun-Yue Miao, Yu Zhang, Ling Shan, Jun-Yan Feng, Fei-Yong Jia, Lin Du

**Affiliations:** Department of Developmental and Behavioral Pediatrics, The First Hospital of Jilin University, Changchun, China

**Keywords:** environmental factors, neurodevelopmental disorder, sedentary behavior, screen time, 25-hydroxyvitamin D

## Abstract

**Objective:**

To analyze the risk factors for developmental quotients (DQs) of children with autism spectrum disorder (ASD) and to better understand the effects of screen time on neurodevelopment in children with ASD.

**Methods:**

We retrospectively analyzed the data of 382 children with ASD, including demographic profiles; socioeconomic status; score on the Chinese parent–child interaction scale (CPCIS); screen time questionnaire; ASD symptom rating scales, including the Autism Behavior Checklist (ABC), Childhood Autism Rating Scale (CARS), and Autism Diagnostic Observation Schedule Second Edition (ADOS-2); and DQs using Griffiths Development Scales–Chinese Edition. Univariate analysis was carried out to analyze the factors related to the DQs of children with ASD, and then the linear regression model was used to identify the independent influencing factors of the DQs of children with ASD.

**Results:**

Vitamin D (β = 0.180, *p* = 0.002), age (β = −0.283, *p* = 0.000) and CARS score (β = −0.347, *p* = 0.000) are risk factors related to DQ of locomotor in children with ASD. Vitamin D (β = 0.108, *p* = 0.034), CARS score (β =  −0.503, *p* = 0.000), ADOS-2 severity score (β =  −0.109, *p* = 0.045) and CPCIS score (β = 0.198, *p* = 0.000) are risk factors related to DQ of personal social skill in children with ASD. Vitamin D (β = 0.130, *p* = 0.018), CARS score (β =  −0.469, *p* = 0.000), and CPCIS score (β = 0.133, *p* = 0.022) are risk factors related to DQ of hearing-speech in children with ASD. Vitamin D (β = 0.163, *p* = 0.003) and CARS score (β =  −0.471, *p* = 0.000) are risk factors related to DQ of eye-hand coordination in children with ASD. Age (β =  −0.140, *p* = 0.020), CARS score (β =  −0.342, *p* = 0.000), ADOS-2 severity score (β = −0.133, *p* = 0.034) and CPCIS score (β = 0.193, *p* = 0.002) are risk factors related to DQ of performance in children with ASD. Vitamin D (β = 0.801, *p* = 0.000) and CPCIS score (β = 0.394, *p* = 0.019) are risk factors related to DQ of practical reasoning in children with ASD.

**Conclusion:**

Vitamin D status, the severity of autistic symptoms and parent-child interaction are risk factors for developmental quotients in children with ASD. Screen exposure time is negatively associated with DQs in children with ASD but is not an independent risk factor for DQs.

## 1. Introduction

Autism spectrum disorder (ASD) is a neurodevelopmental disorder (Battle, 2013) whose main features are social communication dysfunction, restricted interests, and repetitive and stereotypical behaviors. In recent years, the prevalence of ASD has increased. The latest epidemiological survey in the United States showed that the prevalence of ASD was 23 per 1,000 (one in 44) in children aged 8 years (Maenner et al., 2021).

The increasing prevalence of ASD has attracted widespread attention from medical professionals, education professionals, the public, and parents. ASD can impair multiple areas of an individual’s social and cognitive development and may have a different impact depending on the individual’s ability and level of functioning. A high proportion of children with ASD also have neurodevelopmental delays, including those related to not only language and social adaptability but also motor development (Li et al., 2019). Previous studies (Li et al., 2019) have shown that 67.6% of children with ASD who are under 5 years old have combined global developmental delay (GDD), which refers to significant delays in two or more developmental domains [using developmental quotients (DQs) to evaluate neurodevelopment]. Although the term “delay” in GDD seems to suggest the possibility of a maturational catch-up, the reality suggests otherwise. A significant proportion of children with GDD, who are monitored for future DQ scores, may receive a diagnosis of intellectual disability (ID), which suggests that these conditions are closely related and share core clinical features to some degree (Shevell, 2008). Children with ASD combined with ID/GDD show more deficits in social adaptation (Fusar-Poli et al., 2017), which is associated with the long-term prognosis of ASD (Nordin and Gillberg, 1998). Therefore, it is of great value to pay attention to the early neurodevelopment of children with ASD at a young age to consider their prognosis and future social adaptation.

ASD is the result of a combination of environmental and genetic factors (Mandy and Lai, 2016). Considerable evidence has suggested that environmental factors are associated with the development of children with ASD. Research on the effects of electronic screen exposure on child neurodevelopment has been growing in recent years. One study (Carson et al., 2019) showed that screen time was unfavorably associated with social skills throughout early childhood. A recent study published in JAMA Pediatrics (Kushima et al., 2022) showed that among boys, increased screen time at 1 year of age was significantly associated with a diagnosis of ASD at 3 years of age. This suggests a possible association between screen time and the etiology of ASD. Another hot topic of research on environmental risk factors for ASD is the levels of nutritional vitamin D in children with ASD. Vitamin D is an active neurosteroid that plays a crucial neuroprotective role in the developing brain (Ali et al., 2018). In particular, risk factors related to ASD—such as air pollution, cloudy weather, seasonal factors, and migration of dark-skinned immigrants to poleward latitudes—are all associated with vitamin D deficiency (Wang et al., 2022). Current studies have found a low serum vitamin D level in children with ASD, which is negatively correlated with the symptoms of ASD children (Guo et al., 2019; Wang et al., 2020). Prenatal low 25-(OH)D (<20 ng/ml) levels were associated with more autism-related symptoms and behaviors at age 5 (López-Vicente et al., 2019). An animal model of the development of vitamin D deficiency has been proven to reproduce the phenotype related to ASD in the domain of neuroanatomy (Ali et al., 2019). A randomized controlled trial reported that the CARS scores and social intelligence quotients of ASD children after 3 months of supplementation with vitamin D were better than those of controls (Azzam et al., 2015). At present, there is vast evidence supporting the relationship between vitamin D and ASD, including at the animal model, cellular, and physiological levels. However, there is still a lack of evidence from large-sample randomized controlled trials (RCTs), leading to a lack of consensus on the role of vitamin D in children with ASD (Wang et al., 2022).

High screen exposure may cause an insufficient vitamin D status. A study by Solis-Urra et al. (2019) showed that increased sedentary time was associated with vitamin D deficiency. Previous studies by our team have identified inextricable links between electronic screen exposure, vitamin D status, and ASD, and that screen exposure and vitamin D status, as two environmental factors, may play a role in ASD (Wang et al., 2016; Dong et al., 2017; Feng et al., 2017; Dong et al., 2021; Shan et al., 2021). Therefore, it is necessary to focus on the study of vitamin D status and screen time in children with ASD. Indeed, these two environmental factors are not the only environmental factors affecting the neurodevelopment of children with ASD. According to the theory of developmental psychology, the interaction between a child and their caregiver is closely related to child development (Llorca et al., 2017). Other environmental risk factors include sociocultural factors such as family economic conditions, social class, and parental education.

**Figure 1:**
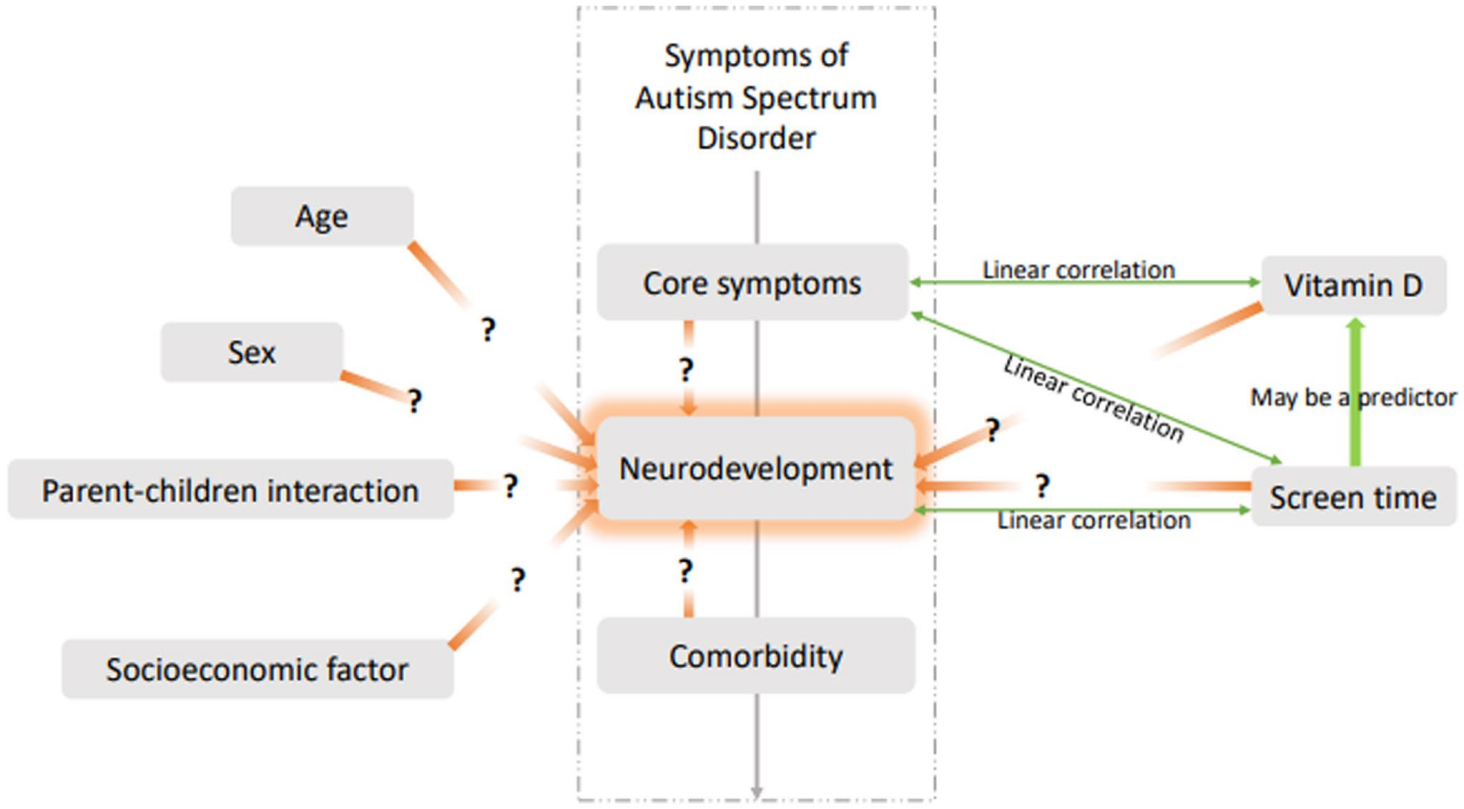
Hypothesis of predictive model for influencing factors of neurodevelopment in children with ASD.

Given the close correlations between electronic screen time, vitamin D status, and neurodevelopment in children with ASD based on previous studies, this study further analyzed the risk factors for DQ scores in children with ASD in China. Other possible factors in our research that may influence DQs in children with ASD include ASD symptoms, comorbidities, and socioeconomic factors. Confounding factors were eliminated to clarify the independent risk factors for DQ scores in children with ASD and better understand the effects of screen time on neurodevelopment in these children (see the hypothesis of a predictive model for the influencing factors of neurodevelopment in children with ASD in Figure 1). With this study, we hope to provide key information for making more accurate intervention decisions in clinical practice.

## 2. Materials and methods

### 2.1. Participants

**Figure 2:**
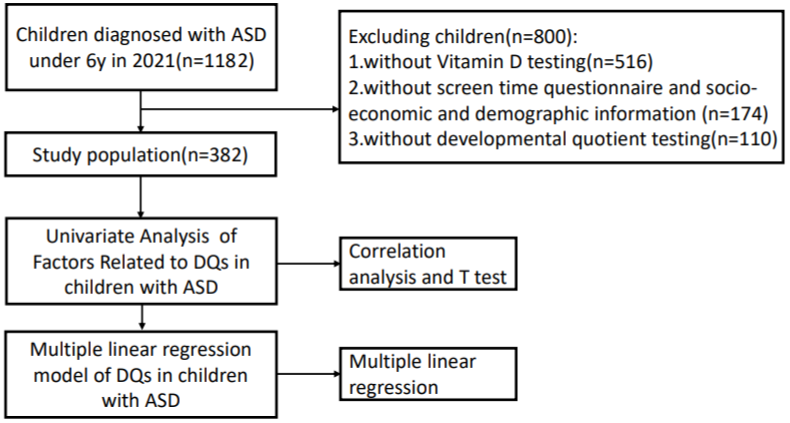
Schematic figure of the study procedure.

Throughout 2021, we retrospectively collected the data of children under 6 years of age who were diagnosed with ASD. All the children (*n* = 1182) were diagnosed according to the DSM-5 criteria and Autism Diagnostic Observation Schedule Second Edition (ADOS-2) for the first time. Developmental and behavioral pediatricians at the First Hospital of Jilin University confirmed that participants were not using high-dose vitamin or mineral supplementation and did not have fragile X syndrome, Rett syndrome, uncontrolled epilepsy, severe dyskinesia, or inherited metabolic diseases. We also excluded children without vitamin D testing (*n* = 516), without a screen time questionnaire score or socioeconomic and demographic information (*n* = 174), and without developmental quotient testing (*n* = 110). A schematic of the study procedure is shown in Figure 2. The ethics committee of our hospital approved this research.

### 2.2. Evaluations and procedure

We retrospectively collected the children’s socioeconomic and demographic information, including age, sex, height, weight, maternal and paternal education levels, main caregivers, household income per year, and vitamin D concentration, from our Outpatient Management Database. We also collected data on the children’s ASD symptoms, neurodevelopment, and parent–child interactions (PCIs) using the Autism Behavior Checklist (ABC), Childhood Autism Rating Scale (CARS), ADOS-2, Griffiths Development Scales-Chinese Edition (GDS-C), and Chinese Parent–Child Interaction Scale (CPCIS). Each enrolled child with ASD completed our screen time questionnaire, which was developed in-house.

The ABC is a 57-item screening checklist for autistic symptoms containing five subscales designed for parent interviews. The CARS consists of 15 subscales, each of which is scored on a continuum from normal to severely abnormal; this required experienced evaluators from our department to observe the behavior of the children in a consulting room. The reliability and validity of the ABC and CARS (Yan et al., 2021) are sufficient. The ADOS-2 (Kamp-Becker et al., 2018), which is utilized as a diagnostic tool for ASD, is a semistructured, standardized assessment that measures ASD symptoms in the domains of social relatedness, communication, play, and repetitive behaviors. It is considered the gold standard for ASD diagnostic evaluation. The GDS-C is a popular tool in the Chinese social context and has good reliability and validity (Li et al., 2020). This tool uses the following five independent subscales to assess the development level of children aged 0–2 years: The locomotor (A scale), personal social skills (B scale), hearing and speech (C scale), eye–hand coordination (D scale), and performance (E scale) subscales. Children aged 3–8 years are also tested to assess their practical reasoning (F scale). The test scores are converted to developmental age (DA) according to the Chinese norm for the GDS-C; the chronological age (CA) is calculated as the date of assessment minus the date of birth; and the development quotients (DQs) = DA × 100/CA (Giagazoglou et al., 2005).

We used a children’s screen time questionnaire that was developed in-house and included screen time on weekdays and weekends, the type of electronic screen (TV, smartphone, computer, etc.), the main activity during screen time (watching cartoons, playing video games, reading Chinese poems, learning, etc.), age at first screen exposure, the use of electronic screens as a tool to raise children (always, sometimes, or rarely), the use of screen time as a reward or punishment (always, sometimes, or rarely), and independent ownership of electronic equipment (yes or no). An evaluator calculated the average daily screen time of the children using the following formula: Average daily screen time (min) = [screen time per day on weekdays (min) × 5 + screen time per day on weekends (min) × 2]/7.

The CPCIS was designed based on the literature and clinical observations in the Chinese context. It is an easily administered, valid, and reliable tool for the assessment of PCIs in Chinese families (Ip et al., 2018). It includes eight items that have good validity and reliability (Ip et al., 2018), including reading, drawing, singing, storytelling, discussing news and affairs, arithmetic and mathematics, English letters, and Chinese characters. Every item receives a score of 0–5 points according to the frequency of interactions between children and their parents per week (representing 0 to 5 times per week).

The serum concentration of vitamin D was detected by high-performance liquid chromatography (HPLC). 25-hydroxyvitamin D (25(OH)D) is the main circulating form of vitamin D. Therefore, we measured the concentration of 25(OH)D to reflect the nutritional status of vitamin D in children with ASD. All samples were tested by the Guangzhou KingMed Diagnostics Group Co., Ltd. (KingMed Diagnostics, SSE 603882).

### 2.3. Statistical analyses

Statistical Package for the Social Sciences (SPSS) software, version 23.0 (SPSS for Windows, SPSS Inc., Chicago, IL, United States), was used to analyze all the data. Continuous variables with normal distributions are represented as the means ± standard deviations (SDs), and categorical variables are represented as frequencies (percentages).

First, we analyzed the correlation among screen time, vitamin D status, age, CARS scores, ADOS-2 severity scores, CPCIS scores, and DQ scores. Correlations were estimated with Pearson’s correlation. Second, we compared the DQ scores of each group (grouped by sex, maternal education level, paternal education level, caregivers, and household income). Continuous variables with normal distributions were compared by the Student’s t test. Then, a multiple linear regression model was used to analyze the factors related to the DQ scores of children with ASD. Factors included in the model were meaningful variables in Pearson’s correlation test and the Student’s t test. The results were considered significant at *p* < 0.05.

## 3. Results

Children with ASD in this study included 291 boys (76.18%) and 91 girls (23.82%), with a sex ratio of 3.2:1. The mean age of the children with ASD was 41.30 ± 12.04 months. The mean concentration of vitamin D was 28.89 ± 13.24 ng/ml, the mean daily electronic screen time was 93.40 ± 107.71 min, and the mean CPCIS score was 17.64 ± 9.26. The mean ABC score of the 392 children with ASD was 56.28 ± 15.50, and the mean CARS score was 34.78 ± 4.19. The mean ADOS-2 severity score was 6.27 ± 1.44 (see details in Table 1).

**Table 1:** Baseline characteristics of participants.

	Children with ASD (*n* = 382)	Boys (*n* = 291, 76.18%)	Girls (*n* = 91, 23.82%)
Age (months)	41.30 ± 12.04	42.00 ± 12.10	39.05 ± 11.60
Vitamin D concentration (ng/ml)	28.89 ± 13.24	29.09 ± 13.44	28.26 ± 12.61
Screen time (mins)	93.40 ± 107.71	85.88 ± 97.15	117.44 ± 133.90
CPCIS score	17.64 ± 9.26	17.49 ± 9.33	18.14 ± 9.02
ABC score	56.28 ± 15.50	56.40 ± 15.34	55.90 ± 16.07
CARS score	34.78 ± 4.19	34.87 ± 4.10	34.49 ± 4.48
ADOS-2 severity score #	6.27 ± 1.44	6.25 ± 1.42	6.33 ± 1.50
Locomotor-DQ	68.66 ± 16.08	68.81 ± 15.78	68.22 ± 17.08
Personal social-DQ	50.29 ± 17.22	50.53 ± 16.96	49.5 ± 17.97
Hearing and speech-DQ	39.05 ± 19.35	38.93 ± 19.44	39.43 ± 19.19
Eye–hand coordination-DQ	55.61 ± 18.47	55.19 ± 18.76	56.91 ± 17.60
Performance-DQ	59.44 ± 20.72	59.03 ± 20.99	60.70 ± 19.96
Practical reasoning-DQ##	70.28 ± 22.04	71.19 ± 18.98	66.56 ± 33.05

#The ADOS-2 severity score was not available for 74 participants (52 boys and 22girls) (ADOS-2 M-T and M-3 have no severity score). ##Practical reasoning-DQ was not available for 324 participants (242 boys and 82girls) (children aged under 3 years are not tested to assess F scale).

Univariate analysis results of the correlation analysis suggested that the average daily screen time of children with ASD was negatively correlated with their Personal social-DQ, Hearing and speech-DQ, and Eye–hand coordination-DQ scores (*r* = −0.142, −0.139, and −0.123, respectively; *p* = 0.006, 0.007, and 0.018, respectively). Vitamin D concentrations in children with ASD were positively correlated with their Locomotor-DQ, Personal social-DQ, Eye–hand coordination-DQ, and Practical reasoning-DQ scores (*r* = 0.224, 0.108, 0.156, and 0.585, respectively; *p* < 0.001, *p* = 0.038, *p* = 0.003, and *p* < 0.001, respectively). Age was negatively correlated with Locomotor-DQ scores (*r* = −0.390, *p* < 0.001) and Practical reasoning-DQ (*r* = −0.431, *p* = 0.003) and positively correlated with Hearing and speech-DQ scores (*r* = 0.194, *p* < 0.001). The CARS score was negatively correlated with all Eye–hand coordination-DQ subscale scores (*r* = −0.156, −0.552, −0.530, −0.448, −0.385, and <0.001, respectively; all *p* < 0.001) except the F scale. The ADOS-2 severity score was negatively correlated with the scores of all subscales of the GDS-C (*r* = −0.183, −0.328, −0.143, −0.286, and −0.273, respectively; *p* = 0.002, <0.001, 0.013, <0.001, and <0.001, respectively) except for the F scale. The CPCIS scores were positively correlated with Personal social-DQ, Hearing and speech-DQ, Eye–hand coordination-DQ, and Performance-DQ scores (*r* = 0.342, 0.275, 0.210, and 0.300, respectively; all *p* < 0.001) (see details in Table 2).

**Table 2:** Correlations among screen time, vitamin D concentration, age, CARS scores, ADOS-2 severity scores, CPCIS scores and DQ scores.

		Locomotor-DQ	Personal social-DQ	Hearing and speech-DQ	Eye–hand coordination-DQ	Performance-DQ	Practical reasoning-DQ
Screen time	*r*	−0.053	−0.142	−0.139	−0.123	−0.046	0.028
	*p*	0.313	0.006**	0.007**	0.018*	0.383	0.596
Vitamin D concentration	*r*	0.224	0.108	0.089	0.156	0.072	0.585
	*p*	<0.001***	0.038*	0.086	0.003**	0.164	<0.001***
Age	*r*	−0.390	0.024	0.194	−0.055	−0.071	−0.431
	*p*	<0.001***	0.645	<0.001***	0.287	0.174	0.003**
CARS score	*r*	−0.156	−0.552	−0.530	−0.448	−0.385	−0.237
	*p*	0.003**	<0.001***	<0.001***	<0.001***	<0.001***	0.126
ADOS-2 severity score	*r*	−0.183	−0.328	−0.143	−0.286	−0.273	0.070
	*p*	0.002**	<0.001***	0.013*	<0.001***	<0.001***	0.648
CPCIS score	*r*	0.097	0.342	0.275	0.210	0.300	0.158
	*p*	0.066	<0.001***	<0.001***	<0.001***	<0.001***	0.293

*p < 0.05, **p < 0.01, and ***p < 0.001.

Univariate analysis results of the Student’s t test showed no difference between boys and girls in any subscale of the GDS-C (all *p* > 0.05). The comparison between children with lower and higher maternal education levels showed no difference in Locomotor-DQ and Practical reasoning-DQ scores, but there was a statistically significant difference in Personal social-DQ, Hearing and speech-DQ, Eye–hand coordination-DQ, and Performance-DQ scores (*t* = −2.102, −4.395, −1.980, and −2.897, respectively; *p* = 0.036, <0.001, 0.049, and 0.004, respectively). The higher the maternal education level of the children with ASD, the better their Personal social-DQ, Hearing and speech-DQ, Eye–hand coordination-DQ, and Performance-DQ scores. The comparison between children with lower and higher paternal education levels showed no difference in Practical reasoning-DQ scores, but there was a statistically significant difference in Locomotor-DQ, Personal social-DQ, Hearing and speech-DQ, Eye–hand coordination-DQ, and Performance-DQ scores (*t* = −2.480, −3.626, −4.927, −2.989, and −3.518, respectively; *p* = 0.014, <0.001, <0.001, 0.003, and <0.001, respectively). The higher the paternal education level of children with ASD, the better their Personal social-DQ, Hearing and speech-DQ, Eye–hand coordination-DQ, and Performance-DQ scores. We divided the children with ASD into two subgroups according to their main caregivers: Children whose main caregivers were their parents and children whose main caregivers were their grandparents or other persons. There was no significant difference in the DQ scores of each subscale between the two groups of children with ASD (all *p* > 0.05). We divided the children with ASD into two subgroups according to annual family income (high and low annual income), and there was no significant difference in the DQ scores of each subscale between the two groups of children with ASD (all *p* > 0.05), except for the Locomotor-DQ score (*t* = −2.691; *p* = 0.008) (see details in Table 3).

**Table 3:** Comparison of DQ scores in each group (grouped by sex, maternal education level, paternal education level, caregivers and household income). Maternal and paternal education levels were defined as higher (junior college or above) or lower (senior high school or below).

		*N* (%)	Locomotor-DQ, M (SD)	Personal social-DQ, M (SD)	Hearing and speech-DQ, M (SD)	Eye–hand coordination-DQ, M (SD)	Performance-DQ, M (SD)	Practical reasoning-DQ, M (SD)
Sex	Boys	291 (76.18)	68.81 (15.78)	50.53 (17.00)	38.93 (19.44)	55.19 (18.76)	59.03 (20.99)	71.19 (19.00)
	Girls	91 (23.82)	68.22 (17.09)	49.55 (17.97)	39.43 (19.20)	56.91 (17.60)	60.70 (20.00)	66.56 (33.05)
	t		0.304	0.471	−0.215	−0.772	−0.67	0.561
	*p*		0.762	0.638	0.83	0.441	0.503	0.578
Maternal education level	Lower	206 (53.93)	67.42 (16/87)	48.49 (17.42)	34.97 (17.65)	53.73 (17.78)	56.44 (20.50)	71 (25.70)
	Higher	176 (46.07)	70.09 (15.13)	52.28 (16.95)	43.70 (20.36)	57.56 (19.16)	62.69 (20.71)	69.97 (20.69)
	*t*		−1.584	−2.102	−4.395	−1.98	−2.897	0.144
	*p*		0.114	0.036*	<0.001***	0.049*	0.004**	0.886
Paternal education Level	Lower	229 (59.95)	66.92 (16.83)	47.64 (17.08)	35.12 (17.01)	53.16 (18.21)	56.28 (20.74)	67.33 (24.84)
	Higher	153 (40.05)	71.16 (14.68)	54.21 (16.83)	45.01 (21.30)	58.99 (18.39)	63.96 (20.03)	71.71 (20.86)
	*t*		−2.48	−3.626	−4.927	−2.989	−3.518	−0.627
	*p*		0.014*	<0.001***	<0.001***	0.003**	<0.001***	0.534
Caregivers	Parents	253 (66.23)	68.26 (16.57)	50.73 (17.22)	38.87 (19.10)	55.69 (18.87)	60.25 (21.09)	68.24 (22.39)
	Grandparents	129 (33.77)	69.70 (15.53)	49.61 (17.67)	39.73 (20.53)	55.31 (18.03)	58.57 (20.24)	76.08 (20.87)
	*t*		−0.778	0.571	−0.388	0.181	0.711	−1.062
	*p*		0.437	0.568	0.698	0.857	0.477	0.294
Household income (per year, 10,000 yuan)	<5	99 (25.92)	64.91 (16.99)	47.89 (18.41)	35.54 (20.31)	53.39 (17.49)	57.16 (20.63)	71.56 (31.66)
	≥5	283 (74.08)	70.04 (15.04)	50.88 (16.49)	39.81 (18.72)	55.62 (18.42)	59.71 (20.84)	71.81 (18.33)
	*t*		−2.691	−1.437	−1.824	−1.012	−1.008	−0.03
	*p*		0.008**	0.152	0.069	0.312	0.314	0.976

*p < 0.05, **p < 0.01, and ***p < 0.001.

Therefore, we entered screen time, vitamin D concentration, age, CARS scores, ADOS-2 severity scores, CPCIS scores, maternal and parental education levels, and household income into the multiple linear regression model to determine whether these covariates had an independent effect on the DQ scores. The categorical variables were coded as follows: Maternal and parental education level (senior high school or below = 0, junior college or above = 1) and household income (<50,000 yuan/year = 0, > 50,000 yuan/year = 1).

The multiple linear regression results showed that vitamin D concentration (*β* = 0.180, *t* = 3.087, *p* = 0.002), age (*β* = −0.283, *t* = −4.678, *p* < 0.001), and CARS scores (*β* = −0.347, *t* = −5.144, *p* < 0.001) were risk factors related to the low Locomotor-DQ scores in children with ASD. The higher the vitamin D concentration, the younger the age, the lower the CARS scores, and the better the Locomotor-DQ scores of children with ASD. Vitamin D concentration (*β* = 0.108, *t* = 2.133, *p* = 0.034), CARS scores (*β* = −0.503, *t* = −8.617, *p* < 0.001), ADOS-2 severity scores (*β* = −0.109, *t* = −1.991, *p* = 0.045), and CPCIS scores (*β* = 0.198, *t* = 3.711, *p* < 0.001) were risk factors related to low Personal social-DQ scores in children with ASD. The higher the vitamin D concentration, the higher the CPCIS scores, the lower the CARS scores, the lower the ADOS-2 severity scores, and the better the Personal social-DQ scores of children with ASD. Vitamin D concentration (*β* = 0.130, *t* = 2.381, *p* = 0.018), CARS scores (*β* = −0.469, *t* = −7.433, *p* < 0.001), and CPCIS scores (*β* = 0.133, *t* = 2.311, *p* = 0.022) were risk factors related to low Hearing and speech-DQ scores in children with ASD. The higher the vitamin D concentration, the higher the CPCIS scores, the lower the CARS scores, and the better the Hearing and speech-DQ scores of children with ASD. Vitamin D concentration (*β* = 0.163, *t* = 2.982, *p* = 0.003) and CARS scores (*β* = −0.471, *t* = −7.430, *p* < 0.001) were risk factors related to low Eye–hand coordination-DQ scores in children with ASD. The higher the vitamin D concentration, the lower the CARS scores, and the better the Eye–hand coordination-DQ scores of children with ASD. Age (*β* = −0.140, *t* = −2.344, *p* = 0.020), CARS scores (*β* = −0.342, *t* = −5.142, *p* < 0.001), ADOS-2 severity scores (*β* = −0.133, *t* = −2.138, *p* = 0.034), and CPCIS scores (*β* = 0.193, *t* = 3.171, *p* = 0.002) were risk factors related to low Performance-DQ scores in children with ASD. The younger the child, the higher the CPCIS score, the lower the CARS score, the lower the ADOS-2 severity score, and the better the Performance-DQ score. Vitamin D concentration (*β* = 0.801, *t* = 5.508, *p* < 0.001) and CPCIS scores (*β* = 0.394, *t* = 2.517, *p* = 0.019) were risk factors related to low Practical reasoning-DQ scores in children with ASD. The higher the vitamin D concentration, the higher the CPCIS scores, and the better the Practical reasoning-DQ scores of children with ASD (see details in Table 4).

**Table 4:** Multiple linear regression model of DQ scores in children with ASD.

		Screen time	Vitamin D	Age	CARS score	ADOS-2 severity score	CPCIS score	Maternal education level	Paternal education level	Household income
Locomotor-DQ	*β*	0.07	0.180	−0.283	−0.347	−0.043	0.041	−0.049	0.106	0.093
	*t*	0.116	3.087	−4.678	−5.144	−0.685	0.671	−0.594	1.282	1.617
	*p*	0.908	0.002**	<0.001***	<0.001***	0.494	0.503	0.553	0.201	0.107
	95%CI	−0.015, 0.017	0.08, 0.363	−0.637, −0.260	−1.816, −0.810	−1.787, 0.865	0.138, 0.280	−6.613, 3.549	−1.843, 8.704	−0.674, 6.855
Personal social-DQ	*β*	−0.078	0.108	−0.070	−0.503	−0.109	0.198	−0.040	0.079	0.009
	*t*	−1.530	2.133	−1.331	−8.617	−1.991	3.711	−0.568	1.095	0.189
	*p*	0.127	0.034*	0.184	<0.001***	0.048*	<0.001***	0.570	0.275	0.850
	95%CI	−0.029, 0.004	0.012, 0.303	−0.325, 0.063	−2.776, −1.743	−2.739, −0.15	0.19, 0.619	−6.727, 3.714	−2.408, 8.428	−3.497, 4.238
Hearing and speech-DQ	*β*	−0.072	0.130	0.030	−0.469	0.062	0.133	0.088	0.052	0.008
	*t*	−1.310	2.381	0.526	−7.433	1.056	2.311	1.143	0.668	0.156
	*p*	0.191	0.018*	0.600	<0.001***	0.292	0.022*	0.254	0.505	0.876
	95%CI	−0.033, 0.007	0.036, 0.386	−0.171, 0.295	−2.962, −1.721	−0.759, 2.514	0.045, 0.560	−2.633, 9.909	−4.303, 8.714	−4.278, 5.014
Eye–hand coordination-DQ	*β*	−0.041	0.163	−0.065	−0.471	−1.111	0.060	−0.058	0.132	−0.031
	*t*	−0.734	2.982	−1.146	−7.430	−1.871	1.029	−0.755	1.700	−0.569
	*p*	0.463	0.003**	0.253	<0.001***	0.063	0.305	0.451	0.090	0.570
	95%CI	−0.025, 0.011	0.081, 0.399	−0.335, 0.089	−2.691, −1.563	−2.900	−0.112, 0.356	−7.881, 3.516	−0.811, 11.017	−5.441, 3.002
Performance-DQ	*β*	0.049	0.073	−0.140	−0.342	−0.133	0.193	0.022	0.089	−0.047
	*t*	0.840	1.266	−2.344	−5.142	−2.138	3.171	0.265	1.086	−0.836
	*p*	0.402	0.207	0.020*	<0.001***	0.034*	0.002**	0.791	0.278	0.404
	95%CI	−0.012, 0.031	−0.068, 0.311	−0.554, −0.048	−2.430, −1.084	−3.702, −0.151	0.017, 0.730	−5.886, 7.719	−3.166, 10.953	−7.179, 2.901
Practical reasoning-DQ	*β*	0.206	0.801	0.008	−0.097	−0.267	0.394	−0.310	0.459	−0.050
	*t*	1.466	5.508	0.058	−0.722	−1.824	2.517	−1.569	2.225	−0.378
	*p*	0.156	<0.001***	0.955	0.477	0.081	0.019*	0.130	0.036*	0.709
	95%CI	−0.032, 0.190	0.881, 1.937	−0.704, 0.744	−2.086, 1.004	−9.134, 0.563	0.169, 1.705	−33.014, 4.499	1.454, 38.820	−15.459, 10.676

*p < 0.05, **p < 0.01, and ***p < 0.001.

## 4. Discussion

Our results mainly suggest that vitamin D concentration, but not electronic screen time, is an independent risk factor for predicting DQ scores in children with ASD. CARS scores and ADOS-2 severity scores for core symptoms of ASD were independent risk factors for DQ scores in children with ASD, and parent–child interaction was also an independent risk factor for DQ scores in children with ASD, but socioeconomic factors were not.

### 4.1. Vitamin D concentration, but not screen time, was a risk factor related to DQ scores in children with ASD

In univariate analysis, Pearson correlation analysis suggested that screen time was negatively correlated with the personal social interaction, hearing–speech, and eye–hand coordination subscale scores of children with ASD. Vitamin D concentration was positively correlated with the locomotor, personal social interaction, eye–hand coordination, and practical reasoning subscale scores. However, correlation analysis did not control for the influence of other variables on the DQ. When we applied the regression model to the analysis, after controlling for other variables, we found that screen time was not an independent factor affecting DQ scores in children with ASD, while vitamin D concentration was an independent factor affecting locomotor, personal social interaction, hearing–speech, hand–eye coordination, and practical reasoning scores in children with ASD.

In early life, increased exposure to screens may deprive children of the opportunity to develop various skills. They may be sedentary in front of a screen, lose opportunities to practice their gross and fine motor skills, and have limited opportunities for interaction and communication, which is detrimental to many skills (Madigan et al., 2019). From this perspective, it seems that screen time may directly influence children’s development. However, our results do not support this. Combined with the results of previous studies, screen time is an independent risk factor for vitamin D deficiency (Shan et al., 2021). We speculate that increased screen time may result in a lower vitamin D concentration, and low vitamin D status is an independent factor for DQ scores in children with ASD.

The second possible explanation is that the effect of screen time on children’s development is delayed. This study was a retrospective study investigating, at the same time, the relationship between electronic screen time and a child’s DQ scores. Increased screen time may predict a child’s future developmental level, not their present level. The results from a longitudinal study published in 2019 by JAMA Pediatrics (Madigan et al., 2019) showed an association between more screen time at 2 years of age and worse developmental performance at 3 years of age, as well as an association between more screen time at 3 years of age and worse developmental performance at 5 years of age. The effect of screen time on the brain development of children with ASD is reflected not only in their DQ scores but also in the onset of ASD. New research shows that screen exposure in boys at 1 year of age is associated with ASD at 3 years of age (Kushima et al., 2022). This may be related to the electromagnetic field of electronic devices. It has been reported that in infants and young children with active neurodevelopment, electromagnetic and light stimulation transmitted through the eyes may affect neurodevelopment (Physics ICoN-IRPJH, 2009; Aldad et al., 2012). This also suggests that the age at which children start to be exposed to electronic screens may also be a factor affecting their development of ASD, which is an interesting question for future research.

### 4.2. Are the core symptoms of ASD risk factors related to low DQ scores in children with ASD?

Many studies have reported an association between children’s cognitive ability and ASD symptoms. Nordin and Gillberg (1998) reported that the cognitive ability of school-aged children is a significant risk factor for ASD. Lord and Schopler (1989) reported that the cognitive assessment of children with ASD is stable, including in children under 3 years of age. We believe that cognitive and social communication impairments may be closely linked, due to their overlapping pathological underpinnings (Kong et al., 2020) related to neurodevelopment. Studies have shown that early experiences of social contact are important for cognitive development (Makinodan et al., 2012), which suggests that lower DQ/IQ scores in children with ASD may be the result of severe social communication deficits. Vivanti et al. (2013) found that children with greater ASD severity at an initial assessment were more likely to present with poorer cognitive outcomes at a later assessment, irrespective of their initial cognitive level, supporting the view that severe ASD symptoms increase the risk of developing ID. Our results suggest that the severity of core symptoms in children with ASD has a strong negative correlation with almost all DQ subscale scores; even after controlling for other variables, the results of the regression analysis remained the same. We also found that in the regression prediction model, among the multiple risk factors for DQ scores on each subscale, the CARS score contributed the most to the DQ score (with the largest absolute value of B in the regression results). The close connection between these factors also suggests that it is important to pay attention to the neuropsychological development of children with ASD and their core symptoms of impaired social communication.

It is also worth noting that ASD symptoms are associated not only with developmental domains related to intellectual development but also with motor ability. Many studies (Ament et al., 2015; de Moraes et al., 2017; Fulceri et al., 2018; Wilson et al., 2018; Masuda et al., 2019) have reported the presence of motor difficulties during the early development of children with ASD. LeBarton et al. (Manwaring et al., 2017) reported that clinical symptoms in children with ASD are positively correlated with vulnerability to impaired motor function during early development. These findings are all consistent with our results.

However, we came to this conclusion while also considering the limitations of the test itself. The GDS-C is a standardized neuropsychological test, and the assessor interacts with and scores the children based on their performance on the test. Compared with the Wechsler Preschool and Primary Scale of Intelligence, although the GDS-C can truly reflect the development of children to a very large extent (Li et al., 2020), children with severe ASD symptoms sometimes have low DQ scores due to poor cooperation, which is a common issue in any neuropsychological test, and some studies suggest that no test is completely suitable for children with ASD (Lord and Schopler, 1989). This may have affected the results to a certain extent, so it is necessary to establish a follow-up cohort and multiple measurements to ensure the stability of the test results.

### 4.3. PCIs are a risk factor related to low DQ scores in children with ASD

Because of the “refrigerator mother theory,” the relationship between parenting and ASD may seem like a taboo topic, but these two issues are completely different. At present, many studies have focused on the important role of PCIs in the healthy development of children. Many parents lack parenting skills for children with ASD characteristics and low social interaction, and these social shortcomings may cause parents to ignore this phenomenon or become highly anxious, which will further cause poor interaction between the parents and their children. This is a vicious cycle in which both parents and children are affected. Such pattern can worsen problem behaviors, anxiety, depression, learning, and neurodevelopment in children. The research of Totsika and Sylva (2004) also shows that child development is related to PCIs. Ronfani et al. (2015) conducted a cohort study to observe the relationship between different domains of children’s neurocognitive development (cognition, language, and motor development) and environmental factors and found that the family environment was the largest variable affecting children’s neurodevelopment. A key point in early childhood development is to observe the interactions between parents and children in the family environment. Appropriate PCIs are an important determinant of cognitive and psychosocial development. The development of the brain involves a combination of genetic and environmental factors, and the early experiences of a child after birth are the result of the continuous interaction of genes and the living environment. Therefore, the care and stimulation provided by caregivers to children in early childhood are critical to their psychosocial development (Walker et al., 2011). Some studies have suggested that socioeconomic factors affect neurodevelopment in multiple ways (Hackman et al., 2010). Socioeconomic status includes many dimensions, such as family income, parents’ occupations, parental educational background, and children’s educational resources. However, our results showed that socioeconomic factors were not independent predictive factors of DQ scores, suggesting that for children with ASD, PCIs may be more relevant as an environmental factor in reshaping children’s neural networks.

Other factors we were interested in included sex. The sex bias in the prevalence of autism has been a topic of interest for several decades, from the perspective of exploring a female-specific protective effect in its prevalence and clinical features (Ruigrok and Lai, 2020). Regarding developmental changes, one study found that diagnosed young girls were more likely to have better cognitive development (Lai and Szatmari, 2020) which is not consistent with our results. We performed univariate analysis, and after grouping by sex, developmental quotients of all GDS-C subscales did not differ between males and females, so sex was not entered into the multiple linear regression model. In the future, we will try to expand the sample size and stratify it by age and other related factors for further discussion.

### 4.4. Limitations and prospects

Since this was a cross-sectional study, it could not show the relationship between current factors and the children’s future neurodevelopment and could not draw causal conclusions. Therefore, longitudinal cohort research is a necessary research direction. The influence of environmental factors on the mechanism of children’s brain development warrants further investigation.

According to the hypothesis of a predictive model for influencing factors of neurodevelopment in children with ASD (Figure 1), we demonstrated that comorbidities may be risk factors affecting the DQ scores of children with ASD. However, we did not retrospectively collect data on common comorbidities, such as attention deficit hyperactivity disorder (ADHD), and we think this is a limitation of our study.

As most ASD samples under consideration did not reach the normal developmental level and can be evaluated as ASD with low DQs, we did not investigate whether the results of this study can be extended to ASD subjects with high DQs. In the future, we hope to collect more children with ASD without developmental delays for further research.

Furthermore, it is important to note that our study focuses on the environmental aspects of ASD, and it is impossible to talk about ASD without mentioning genetic factors. If we overemphasize environmental factors, readers may use our study to justify decisions and recommendations regarding patient care. Our team has also conducted genetic studies, but they have not been published. This study did not combine genetic data with clinical data, which is a limitation.

## 5. Conclusion

Vitamin D status, the severity of autistic symptoms, and parent–child interactions were risk factors for DQ scores in children with ASD, whereas screen exposure time was not. Screen exposure time was negatively associated with DQ scores in children with ASD but was not an independent risk factor for low DQ scores.

## Data availability statement

The raw data supporting the conclusions of this article will be made available by the authors, without undue reservation.

## Ethics statement

The studies involving human participants were reviewed and approved by Ethics Committee of the First hospital of Jilin University. Written informed consent to participate in this study was provided by the participants’ legal guardian/next of kin.

## Author contributions

H-YD and F-YJ: conceptualization. C-YM: methodology and software. YZ and LS: validation and data curation. J-YF: formal analysis. H-YD: investigation and writing—original draft preparation. LD: resources, writing—review and editing, supervision, and project administration. F-YJ: visualization and funding acquisition. All authors contributed to the article and approved the submitted version.

## Funding

All phases of this study were supported by National Natural Science Foundation of China, 81973054; Key Scientific and Technological Projects of Guangdong Province, 2018B030335001 and Natural Science Foundation of Jilin province, 20200201507JC.

## Conflict of interest

The authors declare that the research was conducted in the absence of any commercial or financial relationships that could be construed as a potential conflict of interest.

## Publisher’s note

All claims expressed in this article are solely those of the authors and do not necessarily represent those of their affiliated organizations, or those of the publisher, the editors and the reviewers. Any product that may be evaluated in this article, or claim that may be made by its manufacturer, is not guaranteed or endorsed by the publisher.

## Abbreviations

DQs: Developmental quotients
ASD: Autism spectrum disorder
CPCIS: Chinese parent–child interaction scale
ABC: Autism Behavior Checklist
CARS: Childhood Autism Rating Scale
ADOS-2: Autism Diagnostic Observation Schedule Second Edition
GDS-C: Griffiths Development Scales–Chinese Edition
GDD: Global developmental delay
ID: Intellectual disability
PCIs: Parent–child interactions
HPLC: High-performance liquid chromatography
SPSS: Statistical Product and Service Solutions
